# Validation of the Perform-FES: a new fear of falling scale for hospitalized geriatric patients

**DOI:** 10.1007/s40520-020-01726-6

**Published:** 2020-10-15

**Authors:** Cecilia Ferrer Soler, Clémence Cuvelier, Mélany Hars, François R. Herrmann, Adrienne Charpiot, Catherine Ducharne Wieczorkiewicz, Olivier Bruyère, Charlotte Beaudart, Dina Zekry, Gabriel Gold, Andrea Trombetti

**Affiliations:** 1 Division of Geriatrics, Department of Rehabilitation and Geriatrics, Geneva University Hospitals and Faculty of Medicine, Thônex, Switzerland; 2 Division of Bone Diseases, Department of Medicine, Geneva University Hospitals and Faculty of Medicine, Geneva, Switzerland; 3 Division of Internal Medicine for the Aged, Department of Rehabilitation and Geriatrics, Geneva University Hospitals and Faculty of Medicine, Thônex, Switzerland; 4 Division of Public Health, Epidemiology and Health Economics, World Health Organization Collaborating Center for Public Health Aspects of Musculoskeletal Health and Ageing, University of Liège, Liège, Belgium

**Keywords:** Fear of falling, Assessment, Psychometric validation, Older people, Hospital

## Abstract

**Background:**

Fear of falling is highly prevalent in older adults and associated with numerous negative health events. The main objective of this study was to validate a scale to assess fear of falling, based on performance in real situation (Perform-FES), in a hospitalized geriatric population.

**Methods:**

In this cross-sectional study, 55 patients (mean age: 85.3 years; 58% women) hospitalized in a geriatric hospital in Geneva (Switzerland) were enrolled. The Perform-FES scale was administered to all patients in conjunction with four other fear of falling scales. We determined the floor and ceiling effects, internal consistency, reliability, construct validity, and discriminative power of the Perform-FES scale.

**Results:**

The Perform-FES scale did not demonstrate any significant floor or ceiling effect. It had a good internal consistency (Cronbach’s alpha = 0.78) and an excellent reliability (intraclass correlation coefficient = 0.94). Regarding convergent validity, good correlations were shown between the score obtained on the Perform-FES scale and those obtained on other fear of falling scales. Also, the Perform-FES scale was able to discriminate patients with severe functional impairments (area under the ROC curve = 0.81) and had significantly better discriminating performance than other fear of falling scales.

**Conclusion:**

Findings suggest that the Perform-FES scale has good psychometric properties and may be a relevant tool to assess fear of falling in a geriatric hospitalized population. Future research should focus in particular on assessing the sensitivity to change and the predictive value of this scale in longitudinal studies, and its validity in other populations.

## Introduction

Falls in older people are a common, serious, and growing public health problem. The consequences of this geriatric syndrome can be multiple with an impact on the physical, mental and social well-being of older adults [1–6]. Hence, falls represent the leading cause of fatal and non‐fatal injuries in the population over 65 years of age, and lead to disability, loss of independence, hospitalization, early institutionalization, and premature death [3, 7].

Fear of falling is one of the negative psychological consequences of falling. The notion of fear of falling first appeared in the 1980s. The fear of falling was described in the scientific literature as “post-fall syndrome” or “ptophobia”, a phobic reaction to standing or walking following a fall [8]. Since then, the entity “fear of falling” has evolved, with varied definitions. Especially, fear of falling has been defined as a low perceived self-confidence at avoiding falls during essential activities of daily living, which ultimately leads to restriction of activities [9]. Although this phenomenon is common among older people who fall, it has been estimated that more than 50% of older people expressing a fear of falling have never fallen [10–14]. Furthermore, it has been shown that this feeling of fear of falling is more important among fallers and increases with the number of falls and the severity of their physical consequences [15]. Thus, fall influences the fear of falling and vice versa. The prevalence of fear of falling in the community varies according to studies from 20–85% [16] and from 15–55% [17] for associated avoidance of activity. Fear of falling has been associated with numerous negative health events, including loss of function in relation to restriction of activities of daily living, decreased quality of life, social withdrawal, and with increased risk of admission to long-term care institutions [18].

Many scales are available and validated to assess fear of falling in older people, such as the falls effectiveness scale international (FES-I) [19], the activities-specific balance confidence scale (ABC) [20] and the geriatric fear of falling measurement (GFFM) [21]. Among them, the FES-I has been widely used and studied. This scale measures the degree of confidence the individual feels in performing 16 activities of daily living without falling. This scale showed good psychometric qualities in community samples and has been recommended for clinical and research purposes (e.g., falls prevention research studies) [22]. Given the redundancy of some of the 16 items and in order to reduce the time spent by the clinician on this scale, a shorter version, the Short FES-I, has been validated while preserving good psychomotor properties [23].

Studies having investigated the psychometric qualities of fear of falling scales in the geriatric inpatient population are scarce [24–27]. As fear of falling should be a systematic part of the patient's multifactorial risk assessment, given its impact on patient's rehabilitation as well as functional and social prognosis, a validated scale specifically dedicated for hospitalized older patients is critically required. Improving fear of falling assessment methods in this population should especially enhance identification of patients who would benefit from interventions and foster the development of effective strategies.

A main limitation of most current fear of falling scales is that they are based on answers to a questionnaire—i.e., use of short sentences to state tasks/situations—and may not reflect a person’s feelings during the actual performance of mobility-related tasks/situations [28]. This limitation is especially critical when assessing the fear of falling in hospitalized older patients—who may have some degree of cognitive deficit—whether in the context of a hospitalization for a fall or following an inhospital fall, the most frequent adverse event during hospitalization [29]. Hospitalized older patients, confined in an unusual environment during a hospital stay and with restricted activities, may have difficulties to report their concern about falling in specific daily-life tasks/situations. Also, this limitation may be especially important for patients with cognitive disorders who can often accurately report immediate feelings related to a task or situation but whose responses to a general questionnaire may be highly unreliable.

In view of the limitations discussed above, the objective of this study was to validate a fear of falling scale dedicated to the inpatient geriatric population, based on performance in real situation: the Perform-FES scale. More specifically, this study evaluated the psychometric properties (floor and ceiling effects, validity, reliability, discriminative power) of this new fear of falling scale.

## Materials and methods

### Study design and population

This prospective cross-sectional observational study was conducted in the context of a single-center pilot randomized controlled pilot trial (Hypnosis and Fear of Falling in Seniors: the HYPNOSE trial), carried out in a 296-bed acute care and rehabilitation geriatric hospital of the University Hospitals of Geneva (Switzerland) between January and October 2019. The study included hospitalized patients aged 65 years and older, admitted in a dedicated unit for fall-and-fracture risk assessment and management (“CHutEs et OstéoPoroSe” program) [30]. Patients with a psychiatric history or lacking decisional capacity or who did not speak French were excluded from this study. The sample size was aimed at 50 patients at least for adequate psychometric analysis [31]. The study was approved by the State of Geneva’s Ethics Committee (protocol 2018-01550) and an informed written consent was obtained from all participants before any study-related procedure.

### Measures

#### Perform-FES

The Perform-FES scale is based on the Short FES-I scale. It implies for the patient the performance of seven tasks corresponding to the seven items of the Short FES-I (i.e., describing specific tasks), under the supervision of an occupational therapist, and the assessment of the fear of falling while performing these tasks, including (1) dressing and undressing, (2) showering or bathing, (3) getting up from a chair and sitting down, (4) going up and down stairs, (5) reaching something on the floor, (6) going down and up a slope, and (7) getting out. These tasks were carried out within the patient’s room and hospital’s rehabilitation facilities, including a rehabilitation patio, according to a standardized administration procedure, developed and validated consensually by the project’s interprofessional team prior to the start of the study. When performing each task, the therapist specifically asked the patient about his or her confidence to complete the task without falling. The scoring criteria for each task, based on those of the Short FES-I, were: “Not at all concerned” (1 point), “Somewhat concerned” (2 points), “Fairly concerned” (3 points), and “Very concerned” (4 points). Thus, the total score for this scale ranged from 7 to 28 points.

#### Other measures

Sociodemographic and clinical data were also collected from each patient, including comorbidities (cumulative illness rating scale-geriatric (CIRS-G) [32]), functional performances (short physical performance battery (SPPB) [33]), functional independence (functional independence measure (FIM) [34]), pain (visual analog score (VAS) [35]), cognitive status (mini-mental state examination (MMSE) [36] and clock test [37]), and depression (mini-geriatric depression scale [38]). Self-reported falls during the 6 months preceding the admission were also collected.

The following fear of falling scales were also completed by the patients in their hospital room, randomly before or after the administration of the Perform-FES scale: FES-I [19], Short FES-I [23], ABC-simplified (ABC-S) [20], and GFFM scales [21].

### Analyses

Descriptive statistics were used to characterize the study population and the data were presented as means ± standard deviation or number (percent). Normality was checked using Shapiro–Francia tests. Patient characteristics by sex, and groups according to age and cognitive status, were compared using Chi-square or Fisher’s exact tests for categorical data, and the Mann–Whitney *U* or *t* tests for continuous data. All tests were two-sided, and P values lower than 0.05 were considered significant. STATA version 16.0 software (STATA Corp., College Station, TX, USA) was used for analyses.

The psychometric validation was conducted by examining the floor and ceiling effects, reliability (internal consistency and test–retest reliability), construct validity (convergent and divergent validity), and discriminative power.

#### Floor and ceiling effects

Floor and ceiling effects were considered to be present when a high percentage of the population had the lowest or the highest score, respectively. These effects were considered to be present when more than 20% of patients reached the minimum or maximum score [39].

#### Internal consistency

To measure internal consistency, we used the alpha coefficient of Cronbach. An alpha coefficient value between 0.70 and 0.95 can be considered good and therefore favorable to conclude that the scale is internally consistent [40]. We also assessed the correlation of each item with the total score of the Perform-FES using Spearman’s correlations. A correlation above 0.81 was considered as excellent, between 0.61 and 0.80 as very good, between 0.41 and 0.60 as good, between 0.21 and 0.4 as acceptable, and at last, < 0.20 as insufficient [41].

#### Reliability

To analyze the inter-rater reliability of the Perform-FES, we involved 25 patients evaluated on two occasions (i.e., session 1 and session 2) by 2 different occupational therapists within a maximum interval of 48 h. Sample size was estimated based on Walter et al.’s approximation method [42]. Reliability was assessed using the calculation of intraclass correlation coefficient (ICC) with a 2-way random effects model. Assuming a minimal ICC of 0.5 (po) against a desired of 0.8 (p1), based on *a* = 0.05 and *b* = 0.20, at least 22 patients were required. An ICC over 0.8 was considered as an excellent reliability, according to Landis and Koch’s benchmarks [43].

#### Convergent and divergent validity

To demonstrate convergent validity, we used Spearman’s rho correlation coefficient, assessing the correlation between the scores obtained on the Perform-FES scale and the other fear of falling scales: FES-I, Short FES-I, ABC-S, GFFM. Divergent validity was also assessed by correlating the Perform-FES scale with other scales/tools evaluating a different construct: EVA, CIRS-G, MMSE, clock test, Mini-GDS.

#### Discriminative power

The ability of the Perform-FES to discriminate patients with severe functional limitations (as assessed using the SPPB) was assessed. Univariate and multivariate linear and logistic regression models were used, in particular to determine the odds ratios (OR) and 95% confidence intervals (95% CI). Functional data were treated either as continuous or dichotomous (with a cut-off set to SPPB < 5, patients below this score being at increased risk of inhospital falls and fractures in our setting [44]) variables. In addition, analyses were carried out based on the calculation of the area under the receiver operating characteristic curve (AUC). Finally, we compared the performances of fear of falling scales to discriminate patients with severe functional limitations (SPPB < 5) using a test of equality of the AUCs (“roccomp” procedure in STATA).

## Results

A total of 55 patients were included in this study. The sociodemographic and clinical characteristics of the study population are detailed in Table 1. The mean age of patients was 85.3 ± 6.0 years and 58% (32/55) were female; 58% (32/55) also reported more than one fall in the last 6 months. They presented poor functional performances as revealed by a mean SPPB score of 4.9 ± 2.2, but also cognitive deficits, with a mean MMSE score of 23.0 ± 4.6. Compared with men, women were significantly more likely to report falls over the past 6 months and had higher scores on the Perform-FES scale, but also on other fear of falling scales (*P* < 0.05 for all). The Perform-FES scores did not differ according to age or between patients with or without a MMSE score < 24 (*P* > 0.05 for both).

**Table 1:** Characteristics of patients

Characteristic	Men *N* = 23	Women *N* = 32	Total sample *N* = 55	*P*
Age, years	84.4 ± 4.5	85.8 ± 6.9	85.3 ± 6.0	0.155
BMI, kg/m^2^	25.6 ± 6.2	24.0 ± 5.8	24.6 ± 6.0	0.270
CIRS-G [score range 0–56]	17.5 ± 5.4	14.7 ± 3.2	15.8 ± 4.4	0.008
Falls reported in the last 6 months				
≥ 1 fall	17 (73.9)	32 (100)	49 (89.1)	0.003
≥ 2 falls	8 (34.8)	24 (75.0)	32 (58.2)	
Pain score (visual analog scale) [score range 0–10]	0.8 ± 1.4	1.1 ± 2.1	1.0 ± 1.8	0.783
MMSE [score range 0–30]				0.753
Total score Total score < 24	22.4 ± 5.7 9 (39.1)	23.5 ± 3.7 13 (40.6)	23.0 ± 4.6 22 (40.0)	
Clock test [score range 0–10]	7.2 ± 3.1	6.9 ± 2.9	7.0 ± 3.0	0.569
Mini-GDS [score range 0–4]	1.0 ± 1.2	1.4 ± 1.1	1.2 ± 1.1	0.227
FIM [score range 18–126]	86.5 ± 23.5	92.4 ± 18.0	90.0 ± 20.4	0.454
SPPB [score range 0–12]^a^				
Balance score	2.1 ± 1.1	2.0 ± 1.2	2.1 ± 1.2	0.630
Gait speed score	2.4 ± 1.1	2.1 ± 1.0	2.2 ± 1.0	
Chair stand score	0.5 ± 0.8	0.7 ± 1.0	0.6 ± 1.0	
Total score	5.1 ± 2.2	4.8 ± 2.3	4.9 ± 2.2	
FES-I [score range 16–64]^b^	26.7 ± 9.7	35.2 ± 11.1	32.1 ± 11.3	0.008
Short FES-I [score range 7–28]^b^	11.2 ± 4.3	14.6 ± 5.2	13.3 ± 5.1	0.019
ABC-S [score range 0–45]^c^	28.6 ± 6.5	20.3 ± 7.0	23.4 ± 7.9	< 0.001
GFFM [score range 15–75]^b^	40.9 ± 13.1	47.5 ± 9.1	45.0 ± 11.1	0.021
Perform-FES^d^ [score range 7–28]^b^	9.8 ± 4.6	10.9 ± 2.7	10.4 ± 3.6	0.016

Data presented as mean ± standard deviation or number (percent)

*BMI* body mass index, *CIRS-G *cumulative illness rating scale-geriatric, *MMSE* mini-mental state examination, *Mini-GDS* mini-geriatric depression scale, *FIM* functional independence measure, *SPPB* short physical performance battery, *FES* falls efficacy scale, *ABC-S* activities-specific balance confidence scale-simplified, *GFFFM* geriatric fear of falling measure

^a^This test evaluates three domains of physical function: (1) balance (i.e., ability to stand in three increasingly challenging positions for 10 s each); (2) gait speed (i.e., time to walk 4 m); and (3) lower-limb strength (i.e., time to rise from a chair five times as fast as possible with arms crossed over the chest). Results from each subtest are scored from 0 to 4 and summed, with higher scores indicating higher performance

^b^A higher score indicates a greater concern about falling

^c^A higher score indicates a lower concern about falling

^d^*N* = 52

No adverse event related to the administration of the Perform-FES was reported. The strict administration time range for the Perform-FES was 15–25 min. The Perform-FES could not be administered to 3 of the 55 patients, for logistical reasons related to care planning for two patients and due to the inability to finalize an assessment in one patient.

### Floor and ceiling effects

The minimum and maximum total scores obtained from the Perform-FES scale were 7 and 26 points. All responses were used for each item (i.e., scores from 1 to 4 for each item). The analysis of score distribution revealed an asymmetry in the lowest scores; however, no significant floor effect was found (the proportion of patients reaching the minimum score was 17%). Furthermore, there was no ceiling effect, as no patient obtained the maximum score.

### Internal consistency

The Cronbach’s alpha coefficient of the Perform-FES scale was 0.78. This indicates a high level of internal consistency. The item-total score correlations ranged from 0.45 to 0.71 and were all significantly and positively correlated with the total score of the Perform-FES (*P* < 0.001 for all items) (Table 2). The mean and standard deviation of each item (minimum value = 1, maximum value = 4) are also presented in Table 2.

**Table 2:** Internal consistency: item-total score correlations and Cronbach’s alpha coefficients for the Perform-FES scale

	Mean	Standard deviation	*Correlation item-score total *Coefficient rho	*Correlation item-score* Total *P* value	Alpha coefficient if the item is removed
Item 1	1.43	0.69	0.55	< 0.001	0.75
Item 2	1.51	0.72	0.57	< 0.001	0.76
Item 3	1.32	0.64	0.45	< 0.001	0.78
Item 4	1.81	1.03	0.71	< 0.001	0.74
Item 5	1.38	0.71	0.64	< 0.001	0.72
Item 6	1.40	0.77	0.64	< 0.001	0.74
Item 7	1.63	0.89	0.61	< 0.001	0.75

### Reliability

We found an excellent agreement between the test (mean Perform-FES score at session 1 = 10.2 ± 2.9) and retest (mean Perform-FES score at session 2 = 10.4 ± 3.3) with an ICC of 0.94 (95% CI 0.87–0.97).

### Convergent and divergent validity

**Fig. 1:**
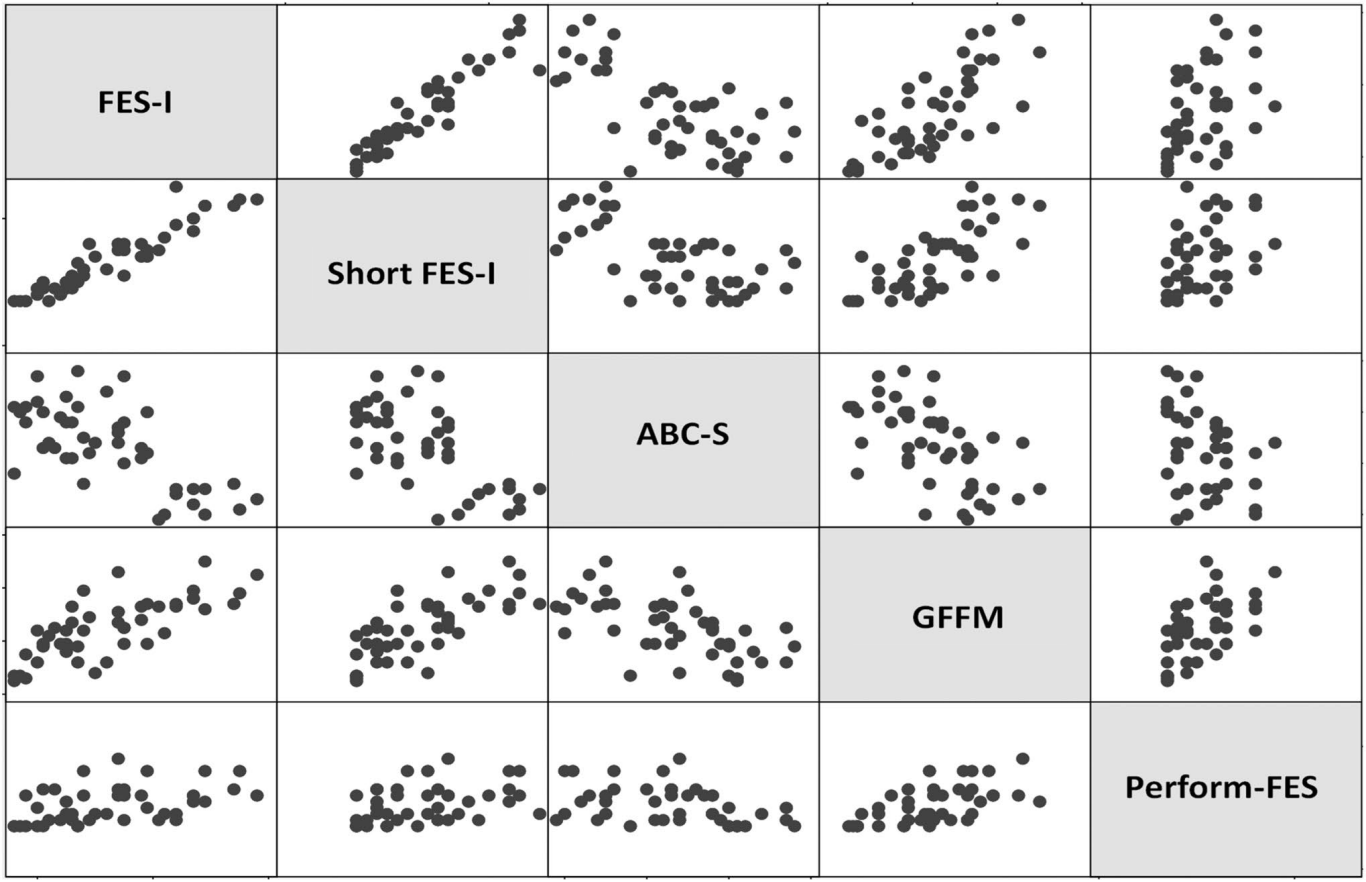
Correlations matrix between scores obtained on the fear of falling scales

Good correlations were observed between the score obtained on the Perform-FES scale and those obtained on the other fear of falling scales evaluating the same construct (*P* < 0.027 for all correlations) (Fig. 1).

For the divergent validity, weak (rho from − 0.08 to 0.21) non-significant correlations were observed between the score obtained on the Perform-FES scale and those obtained on scales/tools evaluating different constructs (VAS, CIRS-G, MMSE, clock test, Mini-GDS; *P* > 0.05 for all correlations).

### Discriminative power

The mean Perform-FES scores were 12.2 ± 3.9 in patients with a SPPB < 5 score and 8.8 ± 2.2 in patients with a SPPB ≥ 5 score (*P* < 0.001). Linear regression models showed a significant association between the Perform-FES score and functional performances (SPPB taken as a continuous variable), even after multiple adjustment on age, sex, MMSE score and CIRS-G score (β = − 0.37; 95% CI − 0.55, − 0.19); *P* < 0.001) (Table 3).

**Table 3:** Associations between the Perform-FES score and functional performances

	Univariate model *β* or OR (95% CI)	Multivariate model 1 *β* or OR (95% CI)^a^	Multivariate model 2 *β* or OR (95% CI)^b^
Perform-FES (SPPB score)	β = − 0.33 (−0.49, −0.18)*	β = − 0.34 (−0.49, −0.18)*	β = − 0.37 (− 0.55, −0.19)*
Perform-FES (SPPB score < 5)	OR = 1.64 (1.21, 2.21)**	OR = 1.65 (1.19, 2.29)**	*OR* = 1.70 (1.19, 2.43)**

Univariate and multivariate linear and logistic regression models with functional data treated either as continuous or dichotomous variables

*SPPB* short physical performance battery, *OR* odds ratio, *CI* confidence interval

^a^Adjusted for age and sex

^b^Adjusted for age, sex, MMSE score and CIRS-G score

^**^P < 0.01

^*^P < 0.001

Logistic regression models showed a significant association between the Perform-FES score and severe functional limitations, even after multiple adjustment (OR = 1.70; 95% CI 1.19, 2.43; *P* < 0.001). The discriminant power of the scale is thereby confirmed (Table 3).

**Fig. 2:**
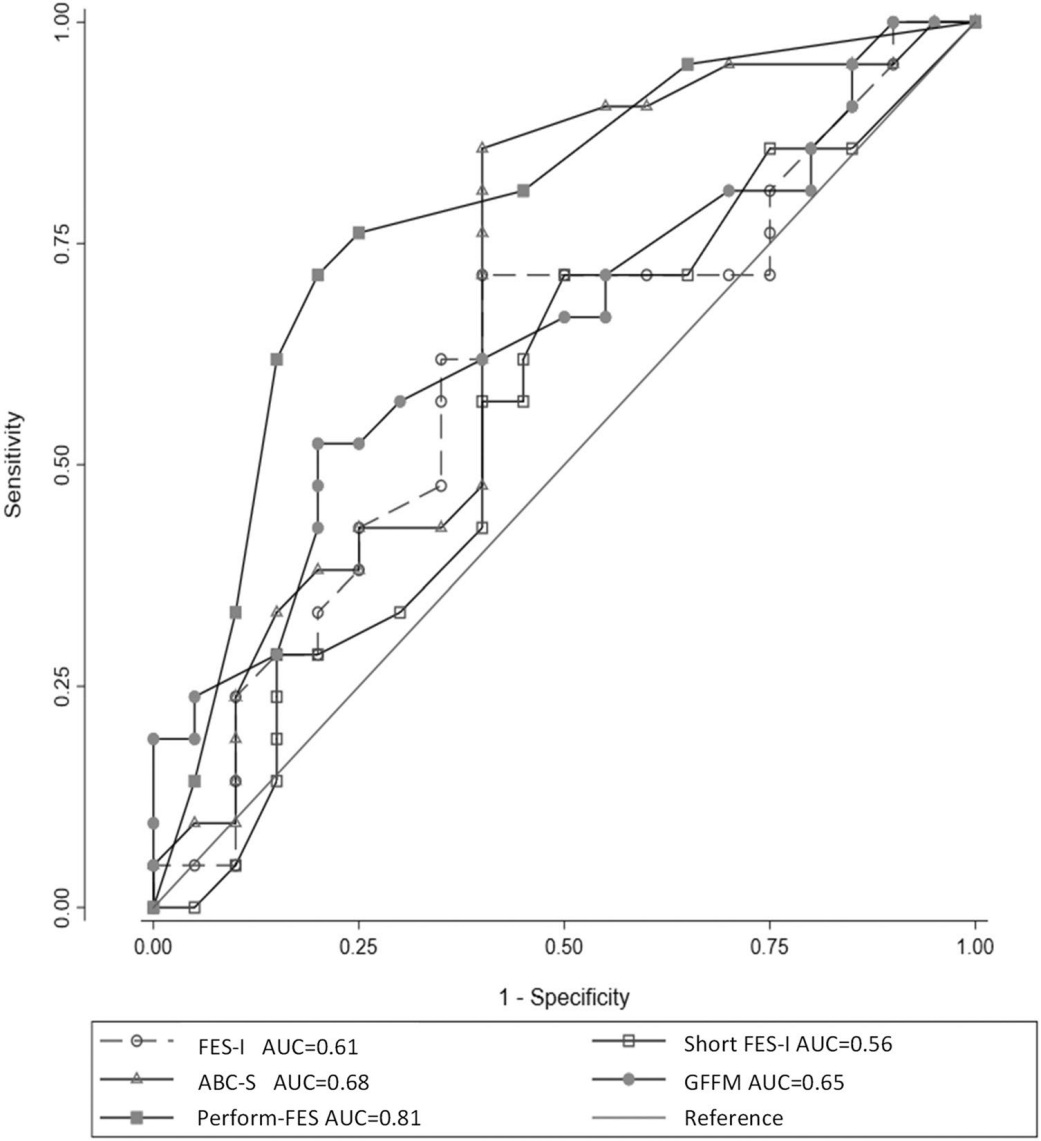
Receiver operating characteristic (ROC) curves for fear of falling scales to discriminate patients with severe functional limitations. Results for models with functional performances treated as dichotomous variable. The diagonal line indicates a reference AUC of 0.50 (no better than chance alone)

Figure 2 presents the receiver operating characteristic (ROC) curves and the area under the ROC curves (AUC) for all fear of falling scales for the discrimination of patients with severe functional limitations (SPPB < 5). The AUC for the Perform-FES scale was 0.81 (95% CI 0.69, 0.94) and the AUC comparison indicated that the Perform-FES scale outperformed other fear of falling scales (*P* < 0.05 for all comparisons).

## Discussion

This study proposes a new way to assess concern about falling in older adults, based on performance in real situation of daily activities, in a hospital setting. The Perform-FES is the first scale specifically designed to measure the degree of concern about falling in this specific environmental context which is the hospital. This first validation study shows that Perform-FES is feasible and has excellent psychometric properties.

The Perform-FES questionnaire had a good internal consistency, with a homogeneity of items within the scale, an excellent reliability, and demonstrated no floor or ceiling effects. The significantly higher scores found in women compared to men on the perform-scale is in line with a large body of data showing a sex influence on fear of falling, with fear of falling found to be more prevalent in women than men [11, 45, 46]. Another reason for this sex difference found in this study might be that women were more likely to have recently fallen.

The convergent validity of the Perform-FES showed good correlation with other fear of falling scales validated in a community population. It should be noted that the correlation between the Perform-FES scale and the Short FES-I scale (which inquiries about fear of falling related to the same tasks but without the patient being put in the situation of actually performing the task) was only 0.45. This suggests that the two assessment methods are similar, but the performance may add meaning [28, 47]. This could be explained by the difficulty patients have in correctly representing the context of each task. This difficulty in conceptualizing activities in a concrete way has already been highlighted during the validation of the Icon-FES scale and the provision of an “unambiguous context” would therefore facilitate this conceptualization [28], especially in the context of cognitive deficits, which may compromise the patients’ abstract abilities [47]. The main advantage of Perform-FES compared to other assessment scales is the performance of activities in the hospital environment.

The Perform-FES scale showed also a significant ability to discriminate patients with severe functional limitations, as assessed by the SPPB, and this independently of age, sex, cognitive impairments and comorbidities. Of note, functional limitations, as assessed by the SPPB, has been shown as an independent predictor of inhospital falls and fractures in our setting [44]. The Perform-FES scale also revealed higher performance than other fear of falling scales in discriminating patients with severe functional limitations. The falls data collection method we used, based on retrospective self-reported fall history, which is prone to bias/underestimate the true occurrence of falls (e.g., because of difficulties placing the event in time, denial), limited our ability to assess the ability of the scale to discriminate fallers. Future longitudinal studies should determine the predictive value of the Perform-FES with respect to prospective incident falls, but also a wide range of negative health events.

In order to properly target interventions in older adults to tackle fear of falling, it is crucial to know a person’s level of fear of falling in different circumstances [48]. Numerous studies have highlighted the value of interventions based on physical exercise or cognitive therapies in the community-dwelling elderly population, whereas studies in the geriatric inpatient population are currently lacking and critically required [49–51]. The sensitivity to change of the Perform-FES scale should be further determined in a longitudinal study, before its utilization to measure the effectiveness and relevance of varied intervention strategies in the hospital setting. Especially, the Perform-FES scale hold promise in the evaluation of an intervention effect in the hospital setting with a short-term period of time frame, in which the subject is obviously not (re)exposed to the situations depicted in the original short-FES and have thus difficulties in evaluating the level of fear the task would be associated.

Although a major strength of this study was that it was conducted under real clinical conditions, several limitations should be acknowledged. First, the patients were older inpatients at high risk for falling enrolled into a fall-and-fracture risk assessment and management program, and the sample size was limited. Thus, the results of the study cannot be generalized to the geriatric inpatient population as a whole and need to be confirmed in larger samples. It is worth noting that patients with cognitive impairments were enrolled in the study, which are frequently excluded from research. A second limitation of the study was the falls data collection method used, based on retrospective self-reported fall history, as detailed above, which limited our ability to assess the ability of the scale to discriminate falls. A final limitation of the study remains the cross-sectional study design used, that precluded addressing the sensitivity to change and predictive value of the Perform-FES scale.

## Conclusions

The Perform-FES is a new way to assess the fear of falling in older hospitalized patients, based on performance in real situation. Findings suggest that this scale, with excellent psychometric properties, may be a relevant tool to assess fear of falling in the hospital setting. Future research should focus in particular on assessing the sensitivity to change and the predictive value of this scale in longitudinal studies, and its validity in other populations and settings.
